# Overfeeding and Hygienic Management

**Published:** 1914-04-11

**Authors:** 


### THE RELATION OF CLOTHES TO APPETITE.

### Overfeeding and Hygienic Management.

Not very long ago (The Hospital, February 21, 1914, p. 558) the views of Dr. Ralph Vincent upon the relative merits of raw milk and pasteurised, or sterilised, milk as infant dietaries were summarised and discussed in these columns. One of the most prominent of those who do not accept the theories and the statistics of Dr. Vincent is Dr. Eric Pritchard. We do not purpose now to quote or to debate Dr. Pritchard's criticisms of the raw milk enthusiasts, but rather to note a very thoughtful and stimulating paper of his* on another aspect of infant feeding.

After a short survey of the theoretical food requirements of a baby, calculated on the quantities of heat dissipated in twenty-four hours by babies of various ages and sizes and on their average daily increase in weight, he points out that such estimates of the required daily diet are faulty and misleading in many particulars. Especially is this the case with the factors of exposure to varying temperature, of fresh air inhaled, of clothing worn, and of exercise taken. A baby in a calorimeter loses a certain number of heat units in a given time, which the calorimeter will register; but it does not at all follow that the same baby would dissipate the same amount of heat if it were not in the equable temperature of the calorimeter. As a matter of fact Lavialle's experiments have shown what a tremendous difference is made to the heat requirements of an infant by the kind of clothing which it wears. Thus a simple baby bonnet saves an infant a heat loss, the milk equivalent of which is two ounces a day. Similarly woollen leggings were proved to save the equivalent of six and a half ounces of milk a day, and the differences between a woollen shawl and a silk one was represented by two and a half ounces.

It will be easily realised that any disquisition on infant feeding which fails to take into account these factors, especially that of the clothing worn, can be satisfactory or adequate. And it follows that the dietetic requirements of two infants, otherwise normal and equal, will be totally different if their environment in respect of clothes, open air, and opportunities for heat dissipation in general are different in the two cases. A slum baby, wrapped in a multiplicity of garments, sleeping in its mother's arms or in her bed, seldom bathed or taken out of doors, has few such opportunities, and hence has an extremely restricted demand for heat production.

These considerations extort from Dr. Pritchard a remarkable confession. "Although in the past," he writes, "I have myself been guilty of drawing up many a table of quantities for the feeding of infants of varying ages, I fully admit now that such tables are worthless unless other important considerations, such as those of clothing, housing, and airing, are simultaneously taken into consideration and allowances made for them." The observations upon which the *optimum* dietaries of infants have been based are those made upon infants living under the best possible hygienic conditions in private houses or in highly efficient institutions. Dr. Pritchard's observations upon slum babies (breast-fed) by means of "test-feeds" have convinced him that such babies take about 33 per cent. less than infants in well-to-do families.

This important discovery Dr. Pritchard then proceeds to interpret; and his explanation of it seems, on the face of things, rational and sensible. He points out that the mortality from diarrhœa and wasting diseases is notoriously much lower among breast-fed babies in the slums than among the bottle fed. Hence it can hardly be supposed that these babies, who take, on the average, so much less food than well-to-do babies, really suffer from starvation. Also the bottle fed, in Dr. Pritchard's experience, do best in the slums upon rations distinctly smaller than those found by experience to fulfil the needs of babies more exposed to loss of heat. He concludes that the slum baby actually requires less food than the other type, because of its restricted chances of dissipating heat; and that a great many slum infants are actually overfed.

Another interesting line of thought considers the defences of an overfed baby. An obvious one is vomiting, and the undue accumulation of fat is another. Less evident are diarrhœa, sweating, dilated vessels on the skin (the cheeks in particular), restlessness, and bronchitis. In other words, the common symptoms of early rickets may be in reality, on this view, attempts of Nature to counteract the evil results of overfeeding. Hedging himself with all possible reservations, and with full knowledge of the scientific worthlessness of any such estimates, Dr. Pritchard nevertheless ventures the statement that no infant, breast fed or artificially fed, can be considered to be in a satisfactory condition unless its physiological demand for food reaches certain approximate standards. Thus an infant of three months, weighing 11 lbs., should require not less than 23 ozs. of breast milk, or its equivalent, in twenty-four hours; one of six months and 16½ lbs. not less than 30 ozs.; one of nine months not less than 36 ozs. If any child taking these quantities (or, of course, more) shows any of the signs of excessive intake just enumerated steps should be taken to increase the demand for food by a revision of the hygienic management of the child. Such a demand can be created by applying the appropriate stimuli of air, light, and cold.

This brief synopsis does scant justice to the lucid arrangement and logical exposition of the original. Those who would follow out in detail Dr. Pritchard's revolutionary hypotheses will be well repaid by a study of the original paper.

---

* "The Food Requirements of Infants." By Dr. Eric Pritchard. *British Journal of Children's Diseases*, February, 1914, p. 49.

